# Biomarker Profiling of Microbial Mats in the Geothermal Band of Cerro Caliente, Deception Island (Antarctica): Life at the Edge of Heat and Cold

**DOI:** 10.1089/ast.2018.2004

**Published:** 2019-12-04

**Authors:** María Ángeles Lezcano, Mercedes Moreno-Paz, Daniel Carrizo, Olga Prieto-Ballesteros, Miguel Ángel Fernández-Martínez, Laura Sánchez-García, Yolanda Blanco, Fernando Puente-Sánchez, Graciela de Diego-Castilla, Miriam García-Villadangos, Alberto G. Fairén, Víctor Parro

**Affiliations:** ^1^ Department of Molecular Evolution, Centro de Astrobiología (CSIC-INTA), Madrid, Spain.; ^2^ Department of Planetology and Habitability, Centro de Astrobiología (CSIC-INTA), Madrid, Spain.; ^3^ Department of Systems Biology, Centro Nacional de Biotecnología, CSIC, Madrid, Spain.; ^4^ Department of Astronomy, Cornell University, Ithaca, New York, USA.

**Keywords:** Deception Island, Cerro Caliente, Geothermal, Microbial mat structure, Microbial metabolism, Biomarker

## Abstract

Substrate–atmosphere interfaces in Antarctic geothermal environments are hot–cold regions that constitute thin habitable niches for microorganisms with possible counterparts in ancient Mars. Cerro Caliente hill in Deception Island (active volcano in the South Shetland Islands) is affected by ascending hydrothermal fluids that form a band of warm substrates buffered by low air temperatures. We investigated the influence of temperature on the community structure and metabolism of three microbial mats collected along the geothermal band of Cerro Caliente registering 88°C, 8°C, and 2°C at the time of collection. High-throughput sequencing of small subunit ribosomal ribonucleic acid (SSU rRNA) genes and Life Detector Chip (LDChip) microarray immunoassays revealed different bacterial, archaeal, and eukaryotic composition in the three mats. The mat at 88°C showed the less diverse microbial community and a higher proportion of thermophiles (*e.g.*, *Thermales*). In contrast, microbial communities in the mats at 2°C and 8°C showed relatively higher diversity and higher proportion of psychrophiles (*e.g.*, *Flavobacteriales*). Despite this overall association, similar microbial structures at the phylum level (particularly the presence of *Cyanobacteria*) and certain hot- and cold-tolerant microorganisms were identified in the three mats. Daily thermal oscillations recorded in the substrate over the year (4.5–76°C) may explain the coexistence of microbial fingerprints with different thermal tolerances. Stable isotope composition also revealed metabolic differences among the microbial mats. Carbon isotopic ratios suggested the Calvin–Benson–Bassham cycle as the major pathway for carbon dioxide fixation in the mats at 2°C and 8°C, and the reductive tricarboxylic acid cycle and/or the 3-hydroxypropionate bicycle for the mat at 88°C, indicating different metabolisms as a function of the prevailing temperature of each mat. The comprehensive biomarker profile on the three microbial mats from Cerro Caliente contributes to unravel the diversity, composition, and metabolism in geothermal polar sites and highlights the relevance of geothermal-cold environments to create habitable niches with interest in other planetary environments.

## 1. Introduction

Geothermal polar environments represent extreme habitats for microbial communities in which thermal adaptation is crucial for their survival and growth (Vogt *et al.*, 1997; Dutta and Chaudhuri, 2010; Reed *et al.*, 2013). Antarctica harbors a combination of glaciers and geothermal activity from which originate ice-free terrains due to underground magma heat (Herbold *et al.*, 2014). These geothermal sites in Antarctica result into hotspots of diversity and, during past glaciations, had a relevant role as long-term refugia with favorable microclimate conditions for microorganisms and plants (Fraser *et al.*, 2014).

Geothermal environments are also hypothesized to be one of the scenarios wherein the origin of life took place on Earth by providing the hydration/dehydration cycle conditions required for molecular self-assembly into membranous compartments (Deamer and Georgiou, 2015), and sustaining the inorganic ion requirements of protocells (Mulkidjanian *et al.*, 2012). Despite the existence of different hypotheses for explaining the origin of life on Earth, such as the sub-marine hydrothermal vents theory (Russell, 2018), the possibility of a geothermal land-based life emergence strengthens the astrobiological relevance of the terrestrial geothermal sites (Van Kranendonk *et al.*, 2017). Therefore, geothermal systems and their associated microbiology on Earth may contribute to decipher extant or extinct life in other planetary bodies (McKay and Stoker, 1989).

At present, Antarctic geothermal environments are associated with four currently active volcanoes: three continental—Mt. Erebus, Mt. Melbourne, and Mt. Rittman; and one maritime— Deception Island (Herbold *et al.*, 2014). Particularly, Deception Island is an active stratovolcano from the South Shetlands Islands (located in the Bransfield Basin) that harbors both extremely cold and hot habitats (Smellie and López-Martínez, 2002). The island is ring shaped with a central flooded caldera and possesses a number of hydrothermal vents due to the interaction of water with hot ascending magmas (Rey *et al.*, 1995; Somoza *et al.*, 2004). Nearly all current geothermal sites in Deception Island are originated from recent past (1967–1970) volcanic events (Baker and McReath, 1971; Rey *et al.*, 1995) and are thus relatively young (Herbold *et al.*, 2014). This recent volcanic activity confers to Deception Island a remarkable environmental value for ecological studies, such as organisms colonization, singularity, and ecological dynamics (Cameron and Benoit, 1970; Barnes *et al.*, 2008), and thus certain sites of the island are comprised within the Antarctic Specially Protected Areas (ASPA) (ATS, 2012).

One of the most relevant ASPA sites in Deception Island is Cerro Caliente, a 107 m high hill (*Cerro*, in Spanish) with a unique microphyte associated with the geothermal activity (ATS, 2012). The geothermal activity in Cerro Caliente comprises a narrow band of substrate affected by hydrothermal fluids ascending from a fault and extending along ca. 40 m length and 3 m wide along the summit ridge (ATS, 2012). Water vapor, carbon dioxide (CO_2_), and minor proportions of hydrogen sulfide and sulfur dioxide, at temperatures that reach up to 100°C, characterize the fumarolic gases and determine the ground geochemistry (Caselli *et al.*, 2004, 2007). Therefore, the co-occurrence of snow and geothermal activity (high temperature and water upwelling) in Cerro Caliente maintains relatively steady liquid water availability over the year (Logan and Allan, 2008) and supplies nutrients to the surface, thus favoring the growth of microbial mats.

Unlike other geothermal areas worldwide (*e.g.*, Miller *et al.*, 2009; Wang *et al.*, 2013; Sharp *et al.*, 2014), the microbial community present in Deception Island is weakly characterized, with few studies focused on the isolation of thermophilic and psychrophilic bacteria (Carrión *et al.*, 2011; Muñoz *et al.*, 2011; Bendia *et al.*, 2018a) with potential use for biotechnological applications (Flores *et al.*, 2018). The coexistence of microorganisms with different thermal and metabolic traits (ammonia oxidation, sulfur reduction, and methanogenesis) was attributed to the steep physicochemical gradients (temperature and geochemical) across the island (Fermani *et al.*, 2007; Bendia *et al.*, 2018b, 2018a). Submarine samples from the flooded caldera also showed coexistence of thermophilic and psychrophilic archaea (Amenábar *et al.*, 2013). Specifically, in the geothermal band of Cerro Caliente, the scarcity of studies on the composition and metabolism of the microbial communities is particularly accentuated. Llarch *et al.* (1997) isolated a thermophilic bacterium from the genus *Bacillus* sp. in the fumarolic water from Cerro Caliente, and Fermani *et al.* (2007) reported the presence of filamentous cyanobacteria and diatoms in microalgal communities growing on the warm substrate. However, little is known about the community structure and metabolisms of the microbial mats thriving in such particular geothermal site.

In this study, the community structure and major metabolic traits were characterized for three microbial mats distributed along the geothermal band of Cerro Caliente. Specifically, we explored the influence of temperature on the bacterial, archaeal, and eukaryotic community structure, and on the carbon and nitrogen metabolism, in three microbial mats registering surface temperatures of 88°C, 8°C, and 2°C at the time of collection and spanned for ca. 10 m long in the Cerro Caliente geothermal summit. To achieve this, we applied isotope-ratio mass spectrometry (IRMS), fluorescence microarray immunoassays, and high-throughput DNA sequencing to investigate the distribution of isotopic (δ^13^C and δ^15^N) and molecular (antibodies and 16S and 18S rRNA genes) biomarkers in the microbial mats.

## 2. Materials and Methods

### 2.1. Field site and sampling

**FIG. 1.**
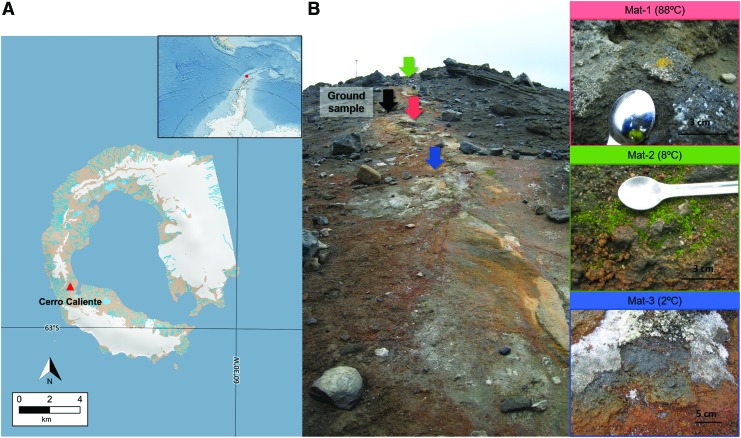
Sampling location at Cerro Caliente, Deception Island. Map showing Deception Island close to the Antarctic Peninsula **(A)**, and pictures showing the sampling sites at Cerro Caliente and the aspect of the microbial mats **(B)**. Black arrow indicates the site for ground sample and color arrows indicate the location for the microbial mat samples. Source of the map: Quantartica (Matsuoka *et al.*, 2018).

Sampling was conducted on February 2, 2012 (*i.e.*, austral summer) in the geothermal summit of Cerro Caliente, a hill located in Deception Island (Fig. 1) formed by pyroclastic deposits of subalkaline andesitic basalt. Three microbial mats with different surface temperatures and extending along ca. 10 m were sampled. The temperature of each mat was measured at 1 cm below the surface with an electronic thermometer, resulting in 88°C for Mat-1, 8°C for Mat-2, and 2°C for Mat-3. Geographical coordinates of Mat-1 and Mat-2 were 62°58′24.6″S and 60°42′44.3″W and those for Mat-3 were 62°58′24.6″S and 60°42′45.1″W. In addition, a ground sample registering 98°C was collected from the subsurface (0–20 cm) at 62°58′25.6″S and 60°42′40.2″W. The three microbial mats and the ground sample were collected using a sterile spatula, introduced in sterile Whirl-Pack sample bags and stored at −20°C until laboratory analyses. Close to the ground sampling spot, a thermocouple was placed at 2.5 cm depth for monitoring the ground temperature every 4 h during a year. The atmospheric temperature was constant during sampling (∼0°C).

### 2.2. Mineralogy and physicochemical analysis of the substrate

The mineralogical characterization of the ground sample was performed with X-ray diffraction (XRD) and infrared spectroscopy techniques. XRD of the powdered ground sample was performed by using a Seifert 3003 TT (GE Inspection Technologies GmbH, Germany) with Cu *K*α anode (λ = 1.542Å). The X-ray generator was set to an acceleration voltage of 40 kV and a filament emission of 40 mA. The range of measurement was from 5° to 60°, with a scanning step size of 0.1°. In addition, the oriented aggregate sample was used to determine the fine grain mineralogy. The powdered sample was also analyzed with a Thermo Nicolet Nexus spectrometer (Nicolet Instrument Corporation, Madison, WI) working with a Diffuse Reflection Praying Mantis (Harrick Scientific, New York, NY), DTGS-KBr detector, and XT-KBr beamsplitter. Measurements were performed with 128 scans and a spectral resolution better than 4 cm^−1^.

Concentration of nitrate (NO_3_^−^), nitrite (NO_2_^−^), phosphate (PO_4_^3−^), and sulfate (SO_4_^2−^) in the ground sample was determined in triplicates by ion chromatography (IC). One gram of substrate was suspended in 12 mL of IC-grade water, vortexed for 1 to 2 min, and then incubated with agitation overnight. Then, the sample was centrifuged (4000 × *g* for 10 min at room temperature) and filtered through a 0.22 μm PTFE filter (Sartorius, Göttingen, Germany) to remove mineral particles. The supernatant was loaded in a Metrohm 861 Advanced Compact Ion Chromatographer (Metrohm AG, Herisau, Switzerland) by an automatic loader and chromatographic separation was performed in a Metrosep A supp 7–250 column (Metrohm AG). The mobile phase consisted of 3.6 mM sodium carbonate with a flow rate of 0.7 mL/min. Quantification of anions was performed by plotting a 6-point calibration curve from each commercial anion standard (Sigma-Aldrich, St. Louis, MO). pH was measured in a suspension of substrate: Milli-Q water (1:2.5) with Eutech pH700 (Thermo Fisher Scientific).

### 2.3. Isotope characterization of the microbial mats

The stable isotopic composition of total organic carbon (TOC) (δ^13^C) and total nitrogen (TN) (δ^15^N) of the bulk microbial mats was measured in triplicates by isotope ratio mass spectrometry (IRMS) with a MAT 253 (Thermo Fisher Scientific) following the analytical methods of the US Geological Survey (Révész *et al.*, 2012). About 0.5 g of dry weight biomass of each mat was grounded and homogenized using a mortar and pestle, and carbonates were removed with the addition of HCl (3M). Then, each mat sample was split into three and replicates were equilibrated for 24 h, adjusted to neutral pH with ultrapure water, and dried in an oven (50°C) until constant weight. The δ^13^C and δ^15^N values were reported in the standard per mil notation using three certified standards (USGS41, IAEA-600, and USGS40) with an analytical precision of 0.1‰. TOC (%) and TN (%) contents were measured during stable isotope measurements using Flash HT Elemental Analyzer (Thermo Fisher Scientific).

### 2.4. DNA extraction of the microbial mats

Genomic DNA of the microbial mats was extracted by using DNeasy PowerBiofilm Kit (QIAGEN, Hilden, Germany) following manufacturer's instructions. Genomic DNA of each microbial mat consisted in two separated extractions of 0.5 g combined at the elution step to increase DNA concentration for sequencing analysis. A negative control of the kit was also performed. DNA concentrations were determined in a NanoDrop ND 1000 spectrophotometer (Thermo Fisher Scientific) and stored at −20°C until sequencing analysis.

### 2.5. 16S rRNA and 18S rRNA genes sequencing and data analysis

Bacterial, archaeal, and eukaryotic communities from microbial mats were identified by the construction of paired-end amplicon libraries on an Illumina MiSeq platform (Illumina Inc., San Diego, CA). Bacterial 16S rRNA V3–V4 hypervariable gene region was amplified with the primer pair 341-F/805-R (Herlemann *et al.*, 2011), archaeal 16S rRNA V2–V3 hypervariable gene region was amplified with the primer pair Arch1F/Arch1R (Cruaud *et al.*, 2014), and eukaryotic 18S rRNA V4–V5 hypervariable gene region was amplified with the primer pair 563F/1132R (Hugerth *et al.*, 2014). Polymerase chain reaction and Illumina MiSeq sequencing were carried out at the Genomic Service in Madrid Science Park Foundation (FPCM).

Raw sequences were processed in MOTHUR software v.1.40.5 (Schloss *et al.*, 2009), using a custom script based on MiSeq SOP (Kozich *et al.*, 2013). In brief, reads below a minimum number of base pairs (≤400 bp for bacteria, ≤300 bp for archaea, and ≤550 bp for eukarya), with ambiguous nucleotide identities and/or homopolymers longer than 8 bp, singletons, and putative chimeras were removed from subsequent analyses. Remaining sequence reads (*i.e.*, 531,261 for bacteria, 295,261 for archaea, and 271,506 for eukarya) were then clustered into operational taxonomic units (OTUs) at the 97% similarity level. Gene library sizes of the mat samples were normalized to the lesser number of sequences (*i.e.*, 124,044 for bacteria, 81,287 for archaea, and 56,561 for eukarya) by random selection. Sequencing depth for each sample was tested by means of rarefaction curves constructed by using iNEXT Online: software for the interpolation and extrapolation of species diversity (Chao *et al.*, 2016).

Taxonomic assignations were performed by comparing OTU's representative sequences with RDP database (RDP reference files v.16; release 11; Cole *et al.*, 2014). OTUs assigned to “cyanobacteria/chloroplast” were further compared with NCBI GenBank, EMBL, Greengenes, and SILVA databases for more precise cyanobacteria taxonomic identification. To avoid false positives, singletons, sequences that were assigned to nonbacterial, nonarchaeal, or noneukaryotic entities in their respective gene libraries, and OTUs that were more represented in the negative controls than in two out of three mat samples, were removed from the analysis. Thus, database screenings discarded 7.5%, 66.2%, and 19.8% of the bacterial, archaeal, and eukaryotic gene libraries, respectively. Raw sequence reads were deposited at the NCBI Sequence Read Archive (SRA) under the BioProject ID PRJNA549255.

### 2.6. Fluorescent sandwich microarray immunoassays with Life Detector Chip

Microbial mats were analyzed by fluorescent sandwich microarray immunoassays (FSMIs) using Life Detector Chip (LDChip), an antibody microarray-based biosensor (Parro *et al.*, 2008; Sánchez-García *et al.*, 2018). The LDChip used in this study contained 187 polyclonal antibodies (purified immunoglobulin G [IgG] fraction) designed to identify bacteria from main phyla (*e.g.*, *Proteobacteria*, *Actinobacteria*, *Firmicutes*, *Bacteroidetes*, and *Cyanobacteria*), endospores and exospores from Gram-positive bacteria and archaea (halophilic, methanogenic, and thermophilic), biological polymers (including lipo/exo-polysaccharides), and conserved proteins and peptides involved in key metabolisms (*e.g.*, nitrogen fixation, nitrogen and sulfur reduction, and iron homeostasis) (Rivas *et al.*, 2008; Parro *et al.*, 2011). The antibodies used in this study are described in Supplementary Table S1. The IgG fraction of each antibody was printed on the surface of epoxy-activated glass slides as described in the study of Blanco *et al.* (2012). For the FSMI, all IgGs were fluorescently labeled with Alexa 647 fluorochrome (Molecular Probes), titrated, and used in a mixture to reveal the immunoreactions, as described in the study of Rivas *et al.* (2008) and Blanco *et al.* (2017).

The procedure for the LDChip analysis was described in detail in the work of Blanco *et al.* (2017). In brief, ∼0.5 g of each mat was resuspended in 2 mL of TBSTRR buffer (0.4 M Tris-HCl pH 8, 0.3 M NaCl, 0.1% Tween 20), ultrasonicated, and filtered through 5 μm filters to be used as a multianalyte-containing sample for the FSMI. LDChip images were analyzed and quantified by GenePix Pro Software (Molecular Devices, Sunnyvale, CA). Final fluorescence intensity (*F*) of each antibody was calculated as described in the study of Rivas *et al.* (2011) and Blanco *et al.* (2012). In addition, a cutoff value was applied to all spots to minimize false positives. This cutoff value was the first interval of *F* with an accumulated frequency >80% and an increase <10%. The output fluorescence data were normalized, attending to the number of positive probes per taxonomic/metabolic group and to total microarray fluorescence values (He *et al.*, 2007).

### 2.7. Statistical analysis

Correspondence analysis (CA) was performed with CANOCO5 v.5.04 software (Microcomputer Power, Ithaca, NY) to explore the relationship between the log-transformed relative abundances of bacterial, archaeal, and eukaryotic taxonomic orders in the three microbial mats. Richness (number of OTUs) and Shannon–Weiner (H′), Simpson, and evenness indices were calculated for the bacterial, archaeal, and eukaryotic community compositions by using the R package “vegan” v.2.4-3 (Oksanen *et al.*, 2017). Dissimilarities between microbial mat communities were estimated using Bray–Curtis distances.

## 3. Results

### 3.1. Mineralogical and physicochemical characterization of the geothermal substrate

**FIG. 2.**
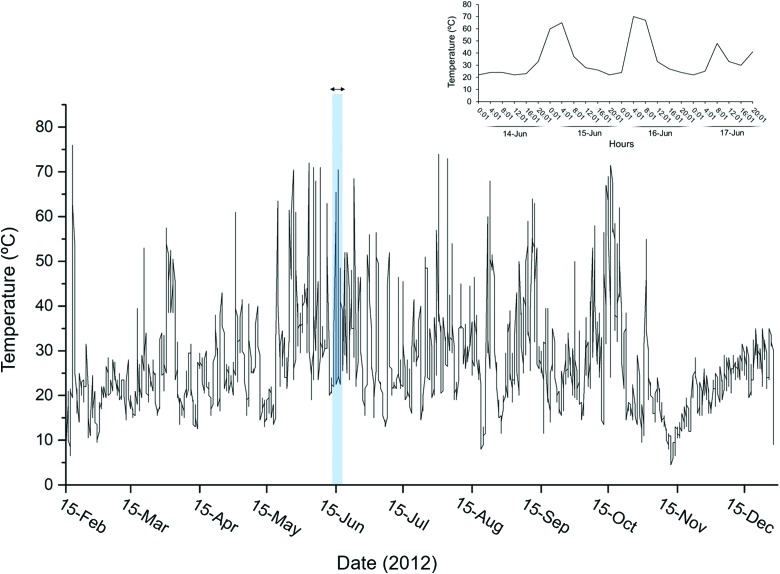
Ground thermal oscillations at 2.5 cm depth in the geothermal band of Cerro Caliente over the year 2012. Temperature was measured with a thermocouple located inside a hole perforated close to the ground sampling point. Upper plot represents a zoom of the temperature oscillations every 4 h recorded from 14 to 17 June.

XRD analysis of the ground sample identified montmorillonite, nontronite, saponite, Na-zeolites, and calcite. The temperature of the ground sample was 98°C, and the pH 7.1. The concentration of NO_3_^−^-N was 0.22 ± 0.15 μg/g and NO_2_^−^-N was under the limit of detection (<1 ppb). Other anions present in the ground sample were soluble PO_4_^3−^-P with 0.29 ± 0.12 μg/g and soluble SO_4_^2−^-S with 1.39 ± 1.17 μg/g. The ground temperature recorded under the surface oscillated from 4.5°C to 76°C over the year and showed daily variations, for example, from 24°C to 70°C on June 15 (Fig. 2). The annual mean, median, and mode temperatures were 27°C, 25°C, and 21°C, respectively.

### 3.2. Carbon and nitrogen elemental and isotopic composition of the microbial mats

**FIG. 3.**
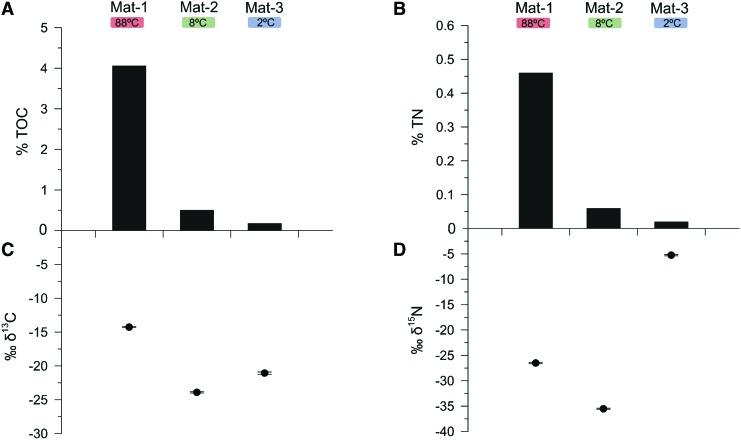
Carbon and nitrogen composition in the three microbial mats. The plots show **(A)** TOC (%) and **(B)** TN (%) contents, and **(C)** isotopic δ^13^C and **(D)** δ^15^N ratios (‰). Error bars indicate standard deviation of triplicates. TN, total nitrogen; TOC, total organic carbon.

TOC and TN concentrations varied markedly between mats. Mat-1 showed the largest TOC (4.1%) and TN (0.5%) concentration (Fig. 3A, B). In contrast, Mat-2 and Mat-3 contained 8-fold and 23-fold lower TOC and TN content than Mat-1, respectively. The lowest TOC (0.18%) and TN (0.02%) contents were observed in Mat-3.

The δ^13^C isotopic composition varied between the three microbial mats (Fig. 3C), with Mat-2 and Mat-3 showing similar depleted δ^13^C ratios (−23.9 ‰ and −21.1 ‰, respectively) in comparison with Mat-1 (−14.3 ‰). For δ^15^N, the most enriched ratio was observed in Mat-3 (−5.3 ‰), in contrast to the depleted signatures of Mat-2 (−35.5 ‰) and Mat-1 (−26.5 ‰) (Fig. 3D).

### 3.3. Bacterial, archaeal, and eukaryotic community structure of the microbial mats

A total of 531,261, 295,261, and 271,506 high-quality sequence reads were recovered from the three mat samples with the bacterial, archaeal, and the eukaryotic SSU rRNA gene amplicons, respectively. After subsampling, control screening and removal of sequences assigned to nonbacterial, nonarchaeal, or noneukaryotic entities in their respective gene libraries, total OTUs identified at 97% similarity were 1925 for bacteria, 55 for archaea, and 991 for eukarya.

**FIG. 4.**
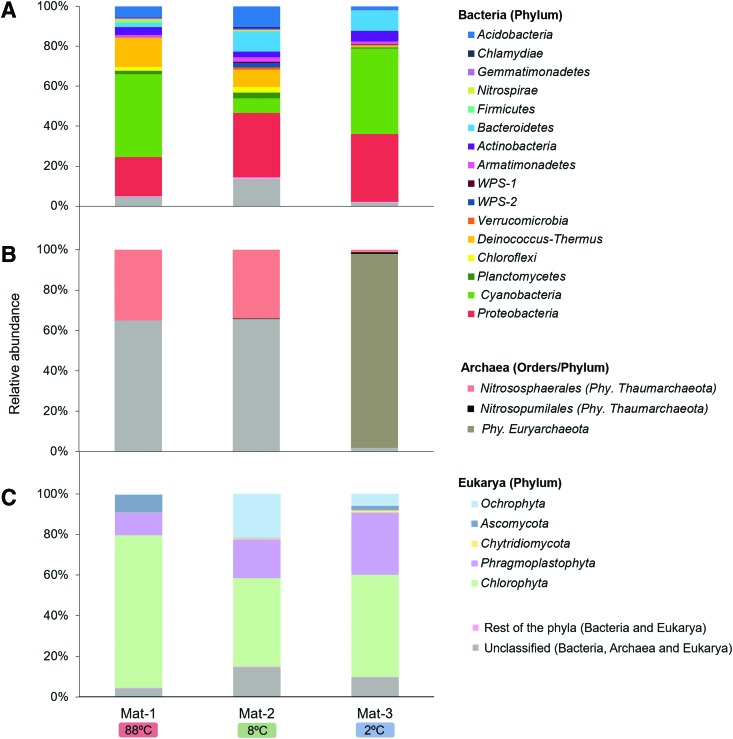
Microbial community structure of the mat samples in the geothermal band of Cerro Caliente. Total bacterial **(A)**, archaeal **(B)**, and eukaryotic **(C)** community composition at phylum level identified in terms of relative abundance. In the Archea domain, the order is also represented. The phyla with relative abundances <0.5% in the three microbial mats are comprised in the “rest of the phyla” group.

The analysis of the bacterial community composition showed 21 distinct phyla, among which 16 exhibited relative abundances >0.5% in at least one mat (Fig. 4A). The three mat profiles shared 95% of the bacterial phyla with differences in their relative abundances. *Cyanobacteria* dominated the taxonomic profiles of Mat-1 (41%) and Mat-3 (43%), and showed a lower proportion in Mat-2 (7%). *Deinococcus-Thermus* accounted for 15% of the total reads in Mat-1, and only accounted for 9% in Mat-2 and 1% in Mat-3. *Bacteroidetes* and *Acidobacteria* also showed differences across mats, ranging from ∼2% to ∼10% in the three microbial mats. *Chloroflexi* was present in similar proportions in Mat-1 (2.1%) and Mat-2 (2.8%), and *Firmicutes* and *Nistrospirae* were mainly identified in Mat-1, with 0.9% each. The phyla *Proteobacteria* was the only phyla present with relatively higher abundance in the three mats (29% ± 8%).

The archaeal community composition in the three microbial mats showed two different phyla, *Thaumarchaeota* and *Euryarchaeota*, and a large fraction of unclassified archaea in Mat-1 (65%) and Mat-2 (66%) (Fig. 4B). The phylum *Thaumarchaeota*, dominated by the order *Nitrososphaerales*, was equally represented in Mat-1 (35%) and Mat-2 (34%), and only accounted for 2% of the total reads in Mat-3. In contrast, the phylum *Euryarchaeota* was abundant in Mat-3 (96%), and it showed lower proportion in Mat-1 and Mat-2 (<0.002%).

The eukaryotic community composition showed 13 different phyla, among which only 5 showed relative abundances >0.5% in at least one of the mats (Fig. 4C). *Chlorophyta* (green algae) and *Phragmoplastophyta* (*Streptophyta*) dominated the three mat profiles and were found in different proportions (44–75% and 11–31%, respectively) across mats. Several fungal taxa were also identified in the three mat samples. *Ascomycota* and *Chytridiomycota* accounted for 9% and 0.11% of the total sequences in Mat-1, and 2% and 1% in Mat-3, respectively. In Mat-2, both phyla were <0.55%. Phylum *Ochrophyta* (diatoms, brown algae, and chrysophytes) accounted for 22% of the total reads in Mat-2.

### 3.4. Correspondence analysis of the composition of the microbial communities

**FIG. 5.**
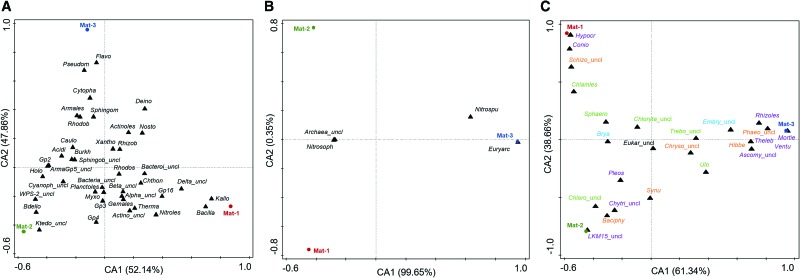
Correspondence Analysis (CA) of the bacterial **(A)**, archaeal **(B),** and eukaryotic **(C)** community composition in Mat-1 (88°C), Mat-2 (8°C), and Mat-3 (2°C) at order level. In absence of order, the upper taxonomic level is shown. For an enhanced view, plot A only shows the 40 bacterial orders with the highest weight (*i.e.*, most frequent). Colors in plot C show the kingdoms of Fungi (purple), Plantae (green algae in green; mosses/other plants in blue), and Protist (brown algae, chrysophytes and amoeba in orange). Acidi, Acidimicrobiales; Actino, Actinobacteria; Actinoles, Actinomycetales; Alpha, Alphaproteobacteria; ArmaGp5, Armatimonadetes Gp5; Armales, Armatimonadales; Ascomy, Ascomycota; Bacilla, Bacillales; Baciphy, Bacillariophytina; Bact, Bacteria; Bacteroi, Bacteroidetes; Basidio, Basidiomycota; Bdello, Bdellovibrionales; Beta, Betaproteobacteria; Brya, Bryales; Burkh, Burkholderiales; Caulo, Caulobacterales; Chlamles, Chlamydomonadales; Chloro, Chlorophyceae; Chlorof, Chloroflexi; Chloryta, Chlorophyta; Chryso, Chrysophyceae; Chthon, Chthonomonadales; Chytri, Chytridiomycota; Conio, Coniochaetales; Cyanoph, Cyanophyceae; Cytopha, Cytophagales; Deino, Deinococcales; Delta, Deltaproteobacteria; Embry, Embryophyta; Eukar, Eukaryota; Euryarc, Euryarchaeota; Flavo, Flavobacteriales; Gemales, Gemmatimonadales; Hibbe, Hibberdiales; Holo, Holophagales; Hypocr, Hypocreales; Ignaviles, Ignavibacteriales; Incert, Incertae; Kallo, Kallotenuales; Krieg, Kriegeriales; Ktedono, Ktedonobacteria; Mortie, Mortierellales; Mucoro, Mucoromycota; Myxo, Myxococcales; Nitroles, Nitrospirales; Nitrosoph, Nitrososphaerales; Nitrospu, Nitrosopumilales; Nosto, Nostocales; Ochro, Ochrophyta; Phaeo, Phaeophyceae; Phragmo, Phragmoplastophyta; Planctoles, Planctomycetales; Pleos, Pleosporales; Pseudom, Pseudomonadales; Rhizob, Rhizobiales; Rhizoles, Rhizophydiales; Rhodob, Rhodobacterales; Rhodos, Rhodospirillales; Saccha, Saccharibacteria; Schizo, Schizoplasmodiida; Sphaero, Sphaeropleales; Sphingob, Sphingobacteriales; Sphingom, Sphingomonadales; Synu, Synurales; Thauma, Thaumarchaeota; Theleb, Thelebolales; Therma, Thermales; Trebo, Trebouxiophyceae; Ulo, Ulotrichales; Ventu, Venturiales; Xantho, Xanthomonadales; _uncl, unclassified. CA, correspondence analysis.

To investigate the relationships between the microorganisms present in the three microbial mats, a CA was performed with the relative abundances of the whole bacterial, archaeal, and eukaryotic data sets at the taxonomic order level of the three microbial mats (Fig. 5).

According to the analyses, most of the bacterial orders showed slight variations in their relative abundances among mats, based on their central location in the plot (Fig. 5A). Still, certain orders showed higher relationship with specific mats. For instance, heat-tolerant bacteria from orders *Kallotenuales*, *Bacillales*, *Thermales*, *Chthonomonadales*, and *Gp16* (phylum *Acidobacteria*) and NO_2_^−^-oxidizing bacteria from the order *Nitrospirales* were more closely associated with Mat-1 (*i.e.*, 88°C) than with the other two mats. In contrast, psychrophilic bacteria from the order *Flavobacteriales* and *Cytophagales* were more related to Mat-3 (*i.e.*, 2°C), as well as *Pseudomonadales*. Unlike this thermal pattern observed in Mat-1 and Mat-3, Mat-2 (*i.e.*, 8°C) showed association with some thermophilic bacteria (*Ktedonobacterales*), in addition to other groups such as anaerobic iron reducers (*Holophagales*) or predators (*Bdellovibrionalles*).

Within the archaeal community, the ammonium-oxidizing order *Nitrosopumilales* and the phylum *Euryarchaeota* were mainly associated with Mat-3 (Fig. 5B). The order *Nitrosophaerales* and unclassified archaea, however, were present in similar relative abundances in both Mat-1 and Mat-2, thus lacking any relationship with a specific mat.

Within the eukaryotic community, several algae, mosses, and unclassified eukaryotes were in similar proportions in all mats, lacking any relationship with a specific mat (Fig. 5C). In contrast, certain orders showed differences across mats. For instance, the cold-tolerant and lichen-associated fungi *Hypocreales* and *Coniochaetales*, the green algae *Chlamydomonadales*, and the amoeba *Shizoplasmodiida* were closely related to Mat-1 (*i.e.*, 88°C). The lichen-associated fungi *Thelebolales*, the yeast *Kriegeriales*, and the cold-tolerant chytrid *Rhizophydiales*, among other fungi, were closely related to Mat-3 (*i.e.*, 2°C), and the fungi *Chytridiomycota* and *LKM15* (phylum *Cryptomycota*), diatoms (*Bacillariophytina*), and green algae (unclassified *Chlorophyceae*) were related to Mat-2 (*i.e.*, 8°C).

### 3.5. Diversity indices

Richness, Shannon–Wiener, and Simpson diversity indices in the three mats were higher for bacteria and eukarya than for archaea (Table 1). Moreover, differences within communities were found across mats. Bacteria showed the highest diversity and richness values in Mat-2 (H′ = 5.24; OTUs = 1214), and the lowest in Mat-1 (H′ = 3.73; OTUs = 845). In eukarya, the highest diversity and richness values were found in Mat-2 (H′ = 2.76; OTUs = 508) and Mat-3 (H′ = 3.30; OTUs = 480), and those for archaea were found in Mat-1 (H′ = 0.84; OTUs = 45) and Mat-2 (H′ = 1.02; OTUs = 41). Evenness indices indicated that Mat-2 possessed the most equally distributed bacterial (evenness = 0.74) and archaeal (evenness = 0.28) community composition, and Mat-3 possessed the most equally distributed eukaryotic community composition (evenness = 0.53).

**Table 1.** Richness (Operational Taxonomic Units), Shannon–Wiener (H′) Diversity, Simpson Diversity, and Evenness Indices from the High-Throughput Sequencing Data of the Bacterial and Archaeal 16S rRNA Genes, and the Eukaryotic 18S rRNA Gene from the Three Microbial Mats

	*Mat-1 (88°C)*	*Mat-2 (8°C)*	*Mat-3 (2°C)*
Bacteria			
OTUs	845	1214	976
H′	3.73	5.24	4.02
Simpson	0.87	0.99	0.93
Evenness	0.55	0.74	0.58
Archaea			
OTUs	45	41	7
H′	0.84	1.02	0.36
Simpson	0.49	0.53	0.13
Evenness	0.22	0.28	0.18
Eukarya			
OTUs	430	508	480
H′	2.02	2.76	3.30
Simpson	0.68	0.87	0.89
Evenness	0.33	0.44	0.53

OTUs = operational taxonomic units.

Bray–Curtis index showed higher similarity in the microbial community structure between Mat-1 and Mat-2 (0.84 for bacteria, 0.58 for eukarya, and 0.37 for archaea) than both mats with Mat-3 (bacteria, archaea, and eukarya >0.90) (Table 2). Still, the three mats shared 15% of the bacterial OTU composition, 6% of the archaeal OTU composition, and 7% of the eukaryotic OTU composition.

**Table 2.** Bray–Curtis Dissimilarity Index from the High-Throughput Sequencing Data of the Bacterial and Archaeal 16S rRNA Genes, and the Eukaryotic 18S rRNA Gene from the Three Microbial Mats

	*Mat-1–Mat-2*	*Mat-1–Mat-3*	*Mat-2–Mat-3*
Bacteria	0.84	0.94	0.90
Archaea	0.37	1.00	1.00
Eukarya	0.58	0.90	0.93

The index ranges from 0 to 1, being 0 when a pair of samples shares the same OTU composition, and 1 when a pair of samples does not share any OTU.

### 3.6. Microbial markers detected by LDChip multiplex immunoassays

**FIG. 6.**
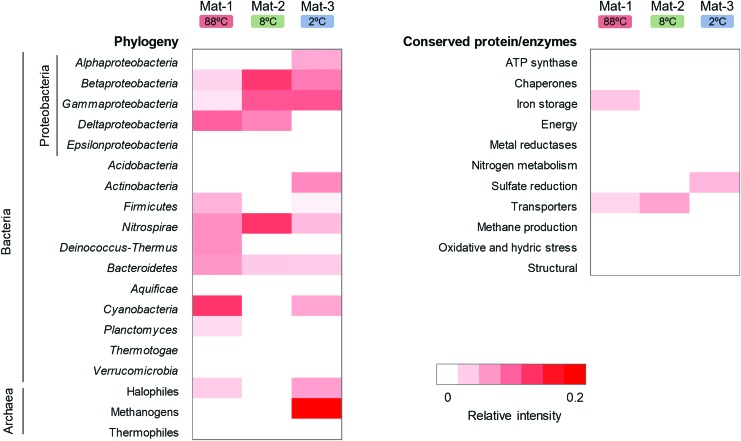
Heatmap of the LDChip immunoassay analysis on the three microbial mats. The antibodies (Supplementary Table S1) were organized into 30 categories based on main phylogenetic groups, metabolic traits, and protein functions. Only phylum Proteobacteria is divided into five taxonomic classes. The averaged fluorescence intensity of the positive signals within each category was used for relative intensity calculation. The color scale represents the relative intensity of the positive signals. White cells stand for values under the detection limit and positive signals are indicated from light pink to red (0.2 as maximum relative intensity). LDChip, Life Detector Chip.

The LDChip detected microbial markers that could be associated with different phylogenetic groups and metabolisms in the mat samples (Fig. 6). For instance, *Cyanobacteria* was immunodetected in Mat-1 and Mat-3, and was negligible in Mat-2. This is in agreement with the 41%, 43%, and 7% of *Cyanobacteria* identified in Mat-1, Mat-3, and Mat-2 with the 16S rRNA gene analysis, respectively. *Proteobacteria* (alpha, beta, gamma, and delta classes) was detected with LDChip in the three mats, and showed the highest relative intensity signals in Mat-2 and Mat-3, consistent with the 32%, 34%, and 20% of relative abundance in Mat-2, Mat-3, and Mat-1 with DNA analyses, respectively. Moreover, *Deinococcus-Thermus* and *Firmicutes* showed the highest relative intensity signals in Mat-1, also coincident with DNA results. *Bacteroidetes* and *Nitrospirae* were immunodetected in the three mats, and *Actinobacteria* was detected in Mat-3. The LDChip also revealed the presence of halophiles in Mat-1 and Mat-3, and methanogens in Mat-3. In addition, LDChip recognized markers from enzymes involved in specific metabolisms such as iron storage and SO_4_^2−^ reduction in Mat-1 and Mat-3, respectively, and protein transporters in both Mat-1 and Mat-2.

## 4. Discussion

### 4.1. Community structure and metabolism in the microbial mats from the geothermal band of Cerro Caliente

The three microbial mats located in the geothermal summit of Cerro Caliente showed differences in their bacterial, archaeal, and eukaryotic community structures below the phylum level. Given the proximity between the three sampling sites and thus their similar mineralogy and geochemistry, other factors, such as temperature, may play a key role on determining their community structures. The selective pressure of temperature on microbial growth has been widely described (Nedwell, 1999; Stetter, 1999, and references herein), and may explain the presence of thermal niches (Clarke, 2017a) in this study. We hypothesize that, in Cerro Caliente, temperature acts as a selective factor that favors preferential growth of specific thermally adapted microbial groups. Indeed, the microbial composition of the hottest mat (*i.e.*, Mat-1) was characterized by a higher proportion of the bacterial orders *Kallotenuales*, *Bacillales*, *Thermales*, and *Chthonomonadales*, which were previously described as heat tolerant or thermophiles (Llarch *et al.*, 1997; Muñoz *et al.*, 2011; Cole *et al.*, 2013; Lee *et al.*, 2014; Bendia *et al.*, 2018a). Contrarily, the coldest mat (*i.e.*, Mat-3) was characterized by a relatively higher proportion of the bacterial orders *Flavobacteriales* and *Cytophagales*, and the fungal order *Rhizophydiales*, previously described as cold tolerant or psychrophiles (Koo *et al.*, 2014; Králová, 2017; Rojas-Jimenez *et al.*, 2017).

Despite the higher proportion of thermal specialists in Mat-1 and Mat-3 than in Mat-2, the three microbial mats shared main phyla (*e.g.*, *Cyanobacteria*, *Proteobacteria*, *Bacteroidetes*, and *Deinococcus-Thermus*) and a proportion of OTUs (15% of bacteria, 6% of archaea, and 7% of eukaryotes). These similarities between mats despite their different temperatures recorded at the time of collection (88°C, 8°C, and 2°C) suggest that water movement along the ground transect and/or ground thermal oscillations may contribute to comparable community structures. Indeed, the daily thermal oscillations of the ground recorded over the year (Fig. 2) indicate that mat temperatures may have been fluctuating, possibly by a mobility of thermal fluids underneath Cerro Caliente and oscillations in the atmospheric temperature.

In a geothermal area, thermal conditions cannot be regarded as static values, but rather dynamic features that typically alternate between activity and inactivity in subsurface fracture systems (Fournier, 1989). Consequently, our study represents a snapshot in time, used to document and compare the microbial community of the three microbial mats at the time of collection but possibly integrating fingerprints from microorganisms that may have been active when the temperatures were within their optimal or tolerance limits for growth. The detection of 75% of green algae and 41% of *Cyanobacteria* in Mat-1 (*i.e.*, 88°C at the time of collection) supports this hypothesis, since the thermal maximum limit for photosynthesis is 73°C (Brock, 1967; Castenholz, 1969). The grayish appearance of Mat-1 (Fig. 1) also supports the inactivity of chlorophyll-bearing microorganisms at the time of collection. Thus, it indicates the preservation of genomic material from photosynthetic microorganisms that were active when lower temperatures allowed photosynthesis to occur. Moreover, the presence of certain thermophiles and psychrophiles in the three mats also supports the influence of the ground thermal dynamics in the composition of the microbial mats. For instance, thermophiles from the order *Ktedonobacterales* (Yabe *et al.*, 2017) were identified in Mat-2 (8°C), and cold-tolerant lichen-associated fungi *Hypocreales* and *Coniochaetales* were present in Mat-1 (88°C) (Park *et al.*, 2015; Zhang *et al.*, 2016). These findings agree with the uneven distribution of thermophiles and psychrophiles in cold and hot soils observed in other locations from Deception Island (Bendia *et al.*, 2018a, 2018b) and suggest that microbial mats in Cerro Caliente can adapt to steep temperature gradients through a heterogeneous microbial composition with different thermal traits. Although geochemical variations at a small spatial scale and their possible influence on the community composition cannot be discarded either (Bendia *et al.*, 2018b), temperature appears to be a more determinant factor modeling the microbial communities in hot springs and other geothermal environments (Wang *et al.*, 2013; Sharp *et al.*, 2014).

The diversity and richness of the microbial mat communities appeared to be also influenced by temperature. The lower diversity and richness values in Mat-1 (*i.e.*, 88°C at the time of collection) as compared with the other two mats (8°C and 2°C) were interpreted as a result of the microbial adaptation to high temperatures and the selection and development of high-temperature specialists in the microbial mat. The influence of temperature on the microbial richness and diversity in geothermal environments was deeply studied in Sharp *et al.* (2014) and concluded that extreme temperatures cause stress and selection of few adapted individuals, thus exerting a strong control on microbial diversity and richness. The low archaeal diversity in the mats from Cerro Caliente as compared with samples from other locations in Deception Island (Signori *et al.*, 2014; Bendia *et al.*, 2018b) may be explained either by the low archaeal evenness or by a dominance of bacteria and eukaryotes in the geothermal band of Cerro Caliente.

The larger TOC and TN values observed in the mat at 88°C (*i.e.*, Mat-1) suggest the influence of temperature on the stimulation of the microbial growth, as temperature is known to increase physiological processes (Clarke, 2017b). The bulk carbon and nitrogen isotopic composition of the microbial mats also evidenced different metabolic traits as a function of temperature. For instance, the relatively enriched δ^13^C ratio observed in Mat-1 suggests CO_2_ fixation pathways mostly related to the reductive tricarboxylic acid (rTCA) cycle (Preuß *et al.*, 1989) and/or the 3-hydroxypropionate (3HP) bicycle (Van Der Meer *et al.*, 2001). This is consistent with the detection of *Chloroflexi*, *Armatimonadetes*, *Nitrospirae*, and *Aquificae* (this one at relative abundance <0.5%) (Herter *et al.*, 2002; Hügler *et al.*, 2007; Hügler and Sievert, 2011; Lee *et al.*, 2014; Alcamán-Arias *et al.*, 2018). The dominance of rTCA or 3HP processes also suggests the lower oxygen availability prevailing under the surface of Mat-1, which is consistent with the generally more oxygen depletion in high-temperature habitats (Hügler and Sievert, 2011) and the presence of facultative/strict anaerobes, such as *Gemmatimonadales* (*e.g.*, *Gemmatimonas*) and *Holophagales* (*e.g.*, *Geothrix*) (Coates *et al.*, 1999; Zhang *et al.*, 2003). In contrast, the relatively depleted δ^13^C isotopic ratios in Mat-2 and Mat-3 revealed a larger implication of the Calvin–Benson–Bassham (CBB) cycle for CO_2_ fixation. This agreed with the presence of bryophytes, green algae, and cyanobacteria in both mats, was coherent with the greener appearance of Mat-2 (Fig. 1), and was congruent with the temperatures measured in both mats at the time of collection (8°C and 2°C), which are compatible with photosynthesis.

Regarding the nitrogen isotopic composition, the wide range of δ^15^N found within the microbial mats (Fig. 3D) reflected distinct participations in the nitrogen cycle. The most enriched δ^15^N ratio in Mat-3 revealed the possible contribution of photosynthetically active cyanobacteria (*e.g.*, order *Nostocales*) and other microorganisms in nitrogen fixation (Rowell *et al.*, 1998). In contrast, the very δ^15^N-depleted signature of Mat-1 and Mat-2 suggests a deep and continuous processing of the organic matter within the nitrogen cycle. Wet and cold ecosystems are known to have δ^15^N-depleted organic values relative to atmospheric N_2_ (*e.g.*, Handley *et al.*, 1999, and references therein). Although the origin of the depleted values is unclear, Handley *et al.* (1999) argued that depleted δ^15^N values could reflect the interaction of internal cycling of nitrogen between live (*e.g.*, active microbial metabolism) and dead organic pools.

According to the microbial composition of the mats, main survival strategies in Cerro Caliente seem to be related to maintain cells active under peaks of extreme high and low temperatures, by combining physiological features and nutritional requirements. Specific lipid membrane composition for thermal stability and ultraviolet resistance (*e.g.*, *Thermales*, Yang *et al.*, 2006), chemolitothrophy (*e.g.*, ammonia oxidation in *Nitrososphaerales*, Stieglmeier *et al.*, 2014), and partition of carbon fixation pathways under high temperatures (*e.g.*, rTCA in *Nitrospirae*, Hügler and Sievert, 2011) are likely the most relevant survival strategies in the mats. The unexpected low relative abundance of the phylum *Firmicutes* may indicate that spore formation represent a low relevant strategy in the geothermal site of Cerro Caliente. In fact, the annual mean temperature was 27°C, which is optimal for the growth of a wide range of microbial phyla. The absence of the phylum *Firmicutes* on the surface of permafrost in Deception Island (Blanco *et al.*, 2012), and on the surface of sediments from fumarole and glacier sites (Bendia *et al.*, 2018b), also supports this hypothesis, since surface cooling or warming may buffer extreme temperatures and cause milder thermal conditions. This is also consistent with the detection of the phylum *Firmicutes* in deeper samples of permafrost (0.6–4.5 m) in Deception Island (Blanco *et al.*, 2012) and those at ∼10 m depth in Alaska permafrost (MacKelprang *et al.*, 2017), where low temperatures were stable over time.

### 4.2. Search for microbial markers with LDChip multiplex immunoassay

Similarities observed between the LDChip and the DNA analyses in the composition of the major bacterial phyla (*e.g.*, *Cyanobacteria*, *Proteobacteria*, and *Deinococcus-Thermus*) highlight the utility of the LDChip for microbial marker detection and determination of the bulk microbial composition of heterogeneous environmental samples. The relationship between immunodetection of methanogens in Mat-3 and the 96% *Euryarchaeota* with DNA analyses (Nichols *et al.*, 2004; Berg *et al.*, 2010) also supports the use of LDChip to get insight into microbial metabolic processes. The detection of methanogens and sulfur reducers in Cerro Caliente is also consistent with the presence of methanogens and sulfur reducers in permafrost and soil samples in other locations of Deception Island (Blanco *et al.*, 2012; Bendia *et al.*, 2018b).

The LDChip is an antibody microarray immunosensor and the core sensing element of Signs Of Life Detector (SOLID), an instrument designed for *in situ* analysis of soil or powdered samples to detect biological polymers and small molecules with interest for planetary exploration (Parro *et al.*, 2005, 2011; Rivas *et al.*, 2008; Blanco *et al.*, 2018; Moreno-Paz *et al.*, 2018). The observed capacity of the LDChip for the detection of molecular markers of the major bacterial phyla in the microbial mats supports SOLID instrument in forthcoming planetary missions such as IceBreaker, aiming to search for biochemical evidence of life in the martian permafrost (McKay *et al.*, 2013; McKay and Parro, 2014). At present, few limitations exist for a thorough analysis of the microbial mat composition with LDChip, including identification of the minor bacterial phyla. Currently, LDChip is under a continuous improvement process by increasing the number of antibodies per phyla and an enhancement of the signal sensitivity to benefit the LDChip performance.

### 4.3. Cerro Caliente as an astrobiological analog for early Mars

Geothermal environments in polar volcanoes constitute a proxy to glimpse into potentially habitable environments beyond Earth, such as Mars and other planetary bodies (Schmidt *et al.*, 2011; Hsu *et al.*, 2015). Our investigations in Cerro Caliente outlined here can serve as an analog of early Mars, when conditions were warmer and wetter than today (Squyres and Kasting, 1994). For instance, the cratered Noachian-aged terrains of Mars are deeply dissected by multiple systems of fluvial valley networks and crater and intercrater lakes (Gulick and Baker, 1989), interpreted as a result from a period of rainfall and surface runoff on early Mars (Craddock and Howard, 2002; Ramirez and Craddock, 2018). This presence of liquid water on the surface, however, is difficult to reconcile with the reduced solar luminosity ca. 3.8 Ga. ago and before (the Sun was ∼25% fainter than today, Spalding *et al.*, 2018). These conditions would have imposed mean temperatures below freezing all over the planet and a climate dominated by generally cold conditions (Fairén *et al.*, 2012). A plausible explanation for flowing liquid water on early Mars is the persistence of glaciovolcanic processes, in which the interaction of volcanic and magmatic activity with surface and subsurface water ice deposits provided liquid water (Smellie and Chapman, 2002; Wilson and Head, 2007; Edwards *et al.*, 2014).

Previous investigations have identified glaciovolcanic features at local scales on Mars, for example, on the flanks of Arsia Mons (Scanlon *et al.*, 2014, 2015), and have been pointed as the origin of local fluvial features. Consequently, it has been suggested that early Mars was affected by the triple point of water during geologically important periods of time, when widespread extensions of soft and deformable ice coexisted with large amounts of water vapor and scarce liquid water. Therefore, glaciomagmatic interactions may have played an important role in volcanic flanks, triggering an active water cycle that altered the landscape and affected the stratigraphy, rock composition, and weathering (Fairén, 2010, 2017).

The evidence of past glaciovolcanic activity promoting fluvial processes on early Mars also allows to hypothesize that Mars had favorable conditions (*e.g.*, liquid water, sources of energy, and nutrients) to trigger processes similar to those that led to the origin of life in our planet (McKay and Stoker, 1989). The polar volcanic environment of Cerro Caliente thus serves as an analog for potential habitable sites on early Mars that could have provided refugia for any developing life (with the common caveats shared by all terrestrial analogs, such as the oxygen availability). The coexistence of evolutionarily distant microorganisms (from *Thaumarchaeota* to *Chlorophyta*) with different thermal tolerances (some adapted to extreme high and cold temperatures) and metabolisms (partition of different CO_2_ fixation pathways) in Cerro Caliente provides diverse molecular biomarker signatures useful to interpret extant or extinct life in current (Mars Science Laboratory) or future (ExoMars and Mars 2020) planetary missions to Mars.

## 5. Conclusions

Microbial mats from Cerro Caliente were assessed with a multianalytical approach to characterize their composition and major metabolisms under variable thermal conditions. The analysis of SSU rRNA genes, multiplex immunoassays, and stable isotope composition evidenced microbial mat composition with different thermal tolerances and metabolisms as a function of the prevailing temperature of each mat. The mat at 88°C showed higher proportion of thermophiles and the rTCA or 3HP cycles as the major carbon fixation pathways, and the mats at 8°C and 2°C showed higher proportion of psychrophiles and the CBB cycle. Beyond this thermal pattern, the three mats shared main microbial phyla, most likely explained by the strong thermal oscillations recorded in the ground over the year. Therefore, thermal factors must be considered to explain the overall microbial community structure and operating metabolisms in Cerro Caliente.

The interest of glaciovolcanic environments as potential habitable sites on early Mars highlights Cerro Caliente as a relevant analog for the study of molecular microbial markers. The correlation between genetic analyses and immunoassays supports the use of the LDChip as a powerful tool for a comprehensive microbial study and its suitability for searching signs of life beyond Earth. Finally, our work stresses on the particularity of Cerro Caliente as a unique habitat with a remarkable environmental value for ecological studies and reinforces its special protection status as ASPA 140.

## Supplementary Material

Supplemental data

## Abbreviations Used

3HP: 3-hydroxypropionate
ASPA: Antarctic Specially Protected Areas
CA: correspondence analysis
CBB: Calvin–Benson–Bassham
CO_2_: carbon dioxide
FSMIs: fluorescent sandwich microarray immunoassays
IC: ion chromatography
IgG: immunoglobulin G
IRMS: isotope ratio mass spectrometry
LDChip: Life Detector Chip
NO_2_^−^: nitrite
NO_3_^−^: nitrate
OTUs: operational taxonomic units
PO_4_^3−^: phosphate
rTCA: reductive tricarboxylic acid
SO_4_^2−^: sulfate
SOLID: Signs Of Life Detector
TN: total nitrogen
TOC: total organic carbon
XRD: X-ray diffraction
