# Supplementary material for: Biomarker Profiling of Microbial Mats in the Geothermal Band of Cerro Caliente, Deception Island (Antarctica): Life at the Edge of Heat and Cold
Source: Astrobiology. 2019 Dec 4;19(12):1490–504. doi: 10.1089/ast.2018.2004 (PMC6918857; doi:10.1089/ast.2018.2004)
Supplement: Supplemental data [file Supp_Table1.doc]

**Appendix Table 1**. List of antibodies printed on LDChip for this study. In “Ab name” column, “A” and “p” indicates protein and pre-immune serum, respectively.

| **No** | **Ab name** | **Source/ Strain** | **Sample/ Culture conditions** | **Immunogen / Fraction** | **References** |
| --- | --- | --- | --- | --- | --- |
|  | **A-1484** | NIFH3 (*Leptospirillum ferrooxidans*) | AKGILKYANSGGVRL | KLH conjugate | Fernández-Calv*o et a*l., 2006 |
|  | **A-1485** | NIFD1 (*Leptospirillum ferrooxidans*) | DFQEKDIVFGGDKKL | KLH conjugate | Fernández-Calv*o et a*l., 2006 |
|  | **A-1490** | FDX2 (*Leptospirillum ferrooxidans*) | HVIITEGFDNLSPME | KLH conjugate | Fernández-Calv*o et a*l., 2006 |
|  | **A-1492** | GLNB2 peptide | VETALRIRTGETGDA | KLH conjugate | Fernández-Calv*o et a*l., 2006 |
|  | **A-1494** | MODA2 (*Leptospirillum ferrooxidans*) | MLAPLHKKIVYANTL | KLH conjugate | Fernández-Calv*o et a*l., 2006 |
|  | **A-1496** | NIFS2 (*Leptospirillum ferrooxidans*) | LPVNKEGRVEIETLK | KLH conjugate | Fernández-Calv*o et a*l., 2006 |
|  | **A-ABCtrans** | ABC transporter (*Thermus scotoductus*) | ABC-transporter protein | Purified recombinant polipeptide | Parr*o et a*l., 2011 |
|  | **A-ApsA-11754** | ApsA (*Desulfovibrio desulfuricans*) | HMMLREMREGRGPIYC | Conjugate | Parr*o et a*l., 2011 |
|  | **A-ASB_11362** | ASB *(Archaeoglobus fulgidus)* | ATP synthase subunit B (Archaea) | Purified recombinant polipeptide | Parr*o et a*l., 2011 |
|  | **A-ASF1_11355** | ASF1 (*Thermotoga maritima*) | ATP synthase F1 alpha subunit | Purified recombinant polipeptide | Parr*o et a*l., 2011 |
|  | **A-BaFER** | Bacterioferritin | Bacterio ferritin | Purified recombinant polipeptide | Riva*s et a*l., 2011 |
|  | **A-BfR** | BFR (*D. desulfuricans*) | CAENFAERIKELFFEP | Conjugate | Parr*o et a*l., 2011 |
|  | **A-BGal** | beta d Galactosidase (E. coli) | Batch culture | Beta d Galactosidase (purified) | BIODESIGN (K03450R) |
|  | **A-CcdA** | CcdA (*Geobacter sulfurreducens*) | CLLGEKRLQVHRKPAGY | Conjugate | Parr*o et a*l., 2011 |
|  | **A-CrReTs_977** | *Thermus scotoductus* | Chromate reductase (membrane) | Purified recombinant polipeptide | Parr*o et a*l., 2011 |
|  | **A-CspA_11357** | CspA (*Pseudomonas putida*) | Cold shock protein A | Purified recombinant polipeptide | Parr*o et a*l., 2011 |
|  | **A-cydA_11758** | CydA (*Shewanella oneidensis*) | YILKKRDLPFARRSFAC | Conjugate | Parr*o et a*l., 2011 |
|  | **A-DhnA1** | DhnA (*Nostoc* PCC 73102) | LRNNAFKQDKDYHLAC | Conjugate | Parr*o et a*l., 2011 |
|  | **A-DhnA2** | DhnA (*Nostoc* PCC 73102) | SGRKAFQRPFEEGVKLC | Conjugate | Parr*o et a*l., 2011 |
|  | **A-DsrA_11365** | DsrA (*Archaeoglobus fulgidus*) | Dissimilatory sulfite reductase | Purified recombinant polipeptide | Parr*o et a*l., 2011 |
|  | **A-DsrB_11368** | DsrB (*Archaeoglobus fulgidus*) | Dissimilatory sulfite reductase | Purified recombinant polipeptide | Parr*o et a*l., 2011 |
|  | **A-Ecoli1** | *Escherichia coli* | E. coli culture | All serotypes | US Biological (E3500-26) |
|  | **A-EFG_11359** | EFG (*Thermotoga. maritima*) | Elongation factor G | Purified recombinant polipeptide | Parr*o et a*l., 2011 |
|  | **A-FdhF** | FDHF (*Escherichia coli* K-12) | Selenopeptide sub. formate DeHase H | Purified recombinant polipeptide | Parr*o et a*l., 2011 |
|  | **A-FeReTs_983** | Iron reductase (*Thermus scotoductus*) | Iron reductase | Purified recombinant polipeptide | Parr*o et a*l., 2011 |
|  | **A-FtsZ** | FtsZ (*Pseudomonas putida*) | CPFEGRKRMQIADEGIR | Conjugate | Parr*o et a*l., 2011 |
|  | **A-GGDEF_11760** | Sensory box/GGDEF Domain | IDLDEFKRIN(DT)FGHKEGDKVC | Conjugate | Parr*o et a*l., 2011 |
|  | **A-GluOxid** | Glucose oxidase | Batch culture (Aspergillus niger) | Glucose oxidase (purified) | BIODESIGN (K59137R) |
|  | **A-GroEL** | GroEL (*Escherichia* coli) | Recombinant | GroEL (purified) | Sigma-Aldrich (G6532) |
|  | **A-Hfer** | Human ferritin | From human liver | Ferritin (purified) | BIODESIGN (H53715) |
|  | **A-Hpylori** | *Helicobacter pylori* | Batch culture (ATCC 43504) | Whole cells | BIODESIGN (B65660R) |
|  | **A-HtpG** | HtpG (*Nostoc* PCC 73103) | HtpG homologous to HSP90 | Purified recombinant polipeptide | Parr*o et a*l., 2011 |
|  | **A-HupS** | HupS (*Leptospirillum ferrooxidans*) | Ni-Fe membrane hydrogenase S chain | Purified recombinant polipeptide | Parr*o et a*l., 2011 |
|  | **A-HyfG** | HyfG (*Escherichia coli*) | Hydrogenase 4 component B | Purified recombinant polipeptide | Parr*o et a*l., 2011 |
|  | **A-ICDH_11755** | ICDH-NAD (*Carboxydothermus hydrogenoformans*) | VL(ES)IKKNKVALKGPC | Conjugate | Parr*o et a*l., 2011 |
|  | **A-IsiA1** | IsiA PSII *(Nostoc* PCC73102) | EISRYKPEIPMGEQGC | Conjugate | Parr*o et a*l., 2011 |
|  | **A-IsiA2** | IsiA PSII *(Nostoc* PCC73102) | FHFEWNDPKLGLILC | Conjugate | Parr*o et a*l., 2011 |
|  | **A-Ktrans_11750** | K+ -transporter (*Geobacter metallireducens*) | LAMSLGRKGEGGTIVC | Conjugate | Parr*o et a*l., 2011 |
|  | **A-Ktrans_11750** | K+ -transporter (Geobacter metallireducens) | LAMSLGRKGEGGTIVC | Conjugate | Parr*o et a*l., 2011 |
|  | **A-Lmono** | *Listeria monocytogenes* | Bacht culture (ATCC 43251) | Whole cells | BIODESING (B65420R) |
|  | **A-McrB_11378** | McrB (*Methanococcoides burtonii*) | Methyl CoM reductase I B subunit | Purified recombinant polipeptide | Parr*o et a*l., 2011 |
|  | **A-Mycob** | *Mycobacterium* sp. | Batch culture M. tuberculosis | Genus-specific antigens extract | BIODESIGN (B47827R) |
|  | **A-NADH_11363** | NuoF (*Pseudomonas putida*) | NADH dehydrogenase I (quinone) | Purified recombinant polipeptide | Parr*o et a*l., 2011 |
|  | **A-NifD_11466** | NifD (*Geobacter metallireducens*) | NifD protein | Purified recombinant polipeptide | Parr*o et a*l., 2011 |
|  | **A-NifD1_12288** | NifD1(*Burkholderia xenovorans*) | VFDKADPDKRPDFVSC | Conjugate | Parr*o et a*l., 2011 |
|  | **A-NifD3_12289** | NifD3 (*Burkholderia xenovorans*) | VFGGDKKLDKIIDEIC | Conjugate | Parr*o et a*l., 2011 |
|  | **A-NifH1_12291** | NifH1 (*Burkholderia xenovorans*) | CNERQTDKELELAEAL | Conjugate | Parr*o et a*l., 2011 |
|  | **A-NifH2_12293** | NifH2 (*Burkholderia xenovorans*) | EFAPESKQAEEYRQLC | Conjugate | Parr*o et a*l., 2011 |
|  | **A-NirS_11369** | NirS (*Pseudomonas aeruginosa*) | Nitrite reductase | Purified recombinant polipeptide | Parr*o et a*l., 2011 |
|  | **A-NOR1_11375** | NOR1 (*Nitrobacter hamburgensis*) | Nitrite oxidoreductase Beta subunit | Purified recombinant polipeptide | Parr*o et a*l., 2011 |
|  | **A-NRA_11912** | NRA (*Geobacter metallireducens*) | Nitrate reductase subunit Alpha | Purified recombinant polipeptide | Parr*o et a*l., 2011 |
|  | **A-OmcS** | OmcS (*Geobacter sulfurreducens*) | CHDPHGKYRRFVDGSI | Conjugate | Parr*o et a*l., 2011 |
|  | **A-OmpA** | OmpA (*Escherichia coli*) | OmpA membrane protein | Purified recombinant polipeptide | Parr*o et a*l., 2011 |
|  | **A-OppA** | OppA (*Bacillus subtilis*) | Oligopeptide binding protein Sub A | Purified recombinant polipeptide | Parr*o et a*l., 2011 |
|  | **A-Paer** | *Pseudomonas aeruginosa* | Batch culture (P. aeruginosa) | Outer membrane protein extract | BIODESIGN (B47578G) |
|  | **A-PfuDPS** | DPS (*Pyrococcus furiosus*) | Fe-binding and storage protein DpS | Purified recombinant polipeptide | Riva*s et a*l., 2011 |
|  | **A-PfuFER** | Ferritin (*Pyrococcus furiosus*) | Ferritin Fe-binding and storage protein | Purified recombinant polipeptide | Riva*s et a*l., 2011 |
|  | **A-PhaC1** | PhaC (*Pseudomonas putida*) | CSLAPDSDDRRFNDPA | Conjugate | Parr*o et a*l., 2011 |
|  | **A-PhaC2** | PhaC (*Pseudomonas putida*) | CLGERAGALKKAPTRL | Conjugate | Parr*o et a*l., 2011 |
|  | **A-PhcA1** | PhcA (*Nostoc* PCC73103) | NTELQSARGRYERAAC | Conjugate | Parr*o et a*l., 2011 |
|  | **A-PhCa2** | PhcA (*Nostoc* PCC73103) | TPGPQFAADSRGKSKC | Conjugate | Parr*o et a*l., 2011 |
|  | **A-PufM1** | PufM (*Rhodospirillum rubrum*) | SRLGGDREVEQITDRC | Conjugate | Parr*o et a*l., 2011 |
|  | **A-PufM2** | PufM (Rhodospirillum *rubrum*) | SRLGGDREVEQITDRC | Conjugate | Parr*o et a*l., 2011 |
|  | **A-RbcL_11374** | RbcL (*Acidithiobacillus ferrooxidans*) | Rubisco large subunit | Purified recombinant polipeptide | Parr*o et a*l., 2011 |
|  | **A-RRO** | Rubredoxin (*Desulfovibrio desulfuricans* G20) | CHTQDETMKALEIKKDV | Conjugate | Parr*o et a*l., 2011 |
|  | **A-Salm** | *Salmonella* sp. (O + H Ags) | Batch culture | Mix S. enteritidis typhym. | BIODESIGN (B65701R) |
|  | **A-SodA** | SodA (*Geobacter sulfurreducens*) | CMLDYGLKRPDYIEAF | Conjugate | Parr*o et a*l., 2011 |
|  | **A-SodF** | SodF (*Geobacter sulfurreducens*) | CARIDKDFGSFDKFKEE | Conjugate | Parr*o et a*l., 2011 |
|  | **A-SsoDPS** | DPS (*Sulfolobus sulfataricus*) | Fe-binding and storage protein Dps | Purified recombinant polipeptide | Riva*s et a*l., 2011 |
|  | **IVE0C_139** | *Leptospirillum ferroxidans* | Batch + Fe^2+^ | Cells sonicated | Parr*o et a*l., 2005 |
|  | **IVE0C_186** | *Leptospirillum ferroxidans* | Batch + Fe^2+^ | Whole cells | Parr*o et a*l., 2005 |
|  | **IVE1BF** | *Leptospirillum pherrifilum* (LPH2) | Fermenter | Biofilm | Riva*s et a*l., 2008 |
|  | **IVE1C1** | *Leptospirillum pherrifilum* (LPH2) | Fermentor | Whole cells | Riva*s et a*l., 2008 |
|  | **IVE1C2** | *Leptospirillum pherrifilum* (LPH2) | Fermenter | Insoluble cell pellet from S100 | Riva*s et a*l., 2008 |
|  | **IVE1S1** | *Leptospirillum pherrifilum* (LPH2) | Fermenter | Culture supernatant | Riva*s et a*l., 2008 |
|  | **IVE1S100** | *Leptospirillum pherrifilum* (LPH2) | Fermenter | Soluble cellular fraction S100 | Riva*s et a*l., 2008 |
|  | **IVE2C1** | *Leptospirillum pherrifilum* spp. | Batch + Fe^2+^ | Whole cells | Riva*s et a*l., 2008 |
|  | **IVE2S1** | *Leptospirillum pherrifilum* spp. | Batch + Fe^2+^ | Culture supernatant | Riva*s et a*l., 2008 |
|  | **IVE2S100** | *Leptospirillum pherrifilum* spp. | Batch + Fe^2+^ | Soluble cellular fraction S100 | Riva*s et a*l., 2008 |
|  | **IVE3C_182** | *Acidthiobacillus ferroxidans* | Batch + Fe^2+^ | Whole cells | Parr*o et a*l., 2005 |
|  | **IVE3C_183** | *Acidthiobacillus ferroxidans* | Batch + Fe^2+^ | Sonicated cells | Parr*o et a*l., 2005 |
|  | **IVE3C1** | *Acidithiobacillus ferrooxidans* | Batch + Fe^2+^ | Whole cells | Riva*s et a*l., 2008 |
|  | **IVE3C2** | *Acidithiobacillus ferrooxidans* | Batch + Fe^2+^ | Insoluble cell pellet from S100 | Riva*s et a*l., 2008 |
|  | **IVE3S1** | *Acidithiobacillus ferrooxidans* | Batch + Fe^2+^ | Culture supernatant | Riva*s et a*l., 2008 |
|  | **IVE3S100** | *Acidithiobacillus ferrooxidans* | Batch + Fe^2+^ | Soluble cellular fraction S100 | Riva*s et a*l., 2008 |
|  | **IVE4C_184** | *Acidithiobacillus thioxidans* | Batch + Fe^2+^ | Whole and sonicated cells | Parr*o et a*l., 2005 |
|  | **IVE4C1** | *Acidithiobacillus thiooxidans* | Batch + S | Whole cells | Riva*s et a*l., 2008 |
|  | **IVE4C1bPfuDPS** | *Acidithiobacillus thiooxidans* | Batch + S | Whole cells | Riva*s et a*l., 2008 |
|  | **IVE4C2** | *Acidithiobacillus thiooxidans* | Batch + S | Insoluble cell pellet from S100 | Riva*s et a*l., 2008 |
|  | **IVE4S100** | *Acidithiobacillus thiooxidans* | Batch + S | Soluble cellular fraction S100 | Riva*s et a*l., 2008 |
|  | **IVE5C1** | *Acidithiobacillus albertensis* | Batch + S | Whole cells | Riva*s et a*l., 2008 |
|  | **IVE5C2** | *Acidithiobacillus albertensis* | Batch + S | Insoluble cell pellet from S100 | Riva*s et a*l., 2008 |
|  | **IVE5S100** | *Acidithiobacillus albertensis* | Batch + S | Soluble cellular fraction S100 | Riva*s et a*l., 2008 |
|  | **IVE6C1** | *Acidithiobacillus caldus* | Batch + S | Whole cells | Riva*s et a*l., 2008 |
|  | **IVE6C2** | *Acidithiobacillus caldus* | Batch + S | Insoluble cell pellet from S100 | Riva*s et a*l., 2008 |
|  | **IVE6S100** | *Acidithiobacillus caldus* | Batch + S | Soluble cellular fraction S100 | Riva*s et a*l., 2008 |
|  | **IVE6S2** | *Acidithiobacillus caldus* | Batch + S | Supernatant from EDTA wash | Riva*s et a*l., 2008 |
|  | **IVE7C1** | *Halothiobacillus neapolitanus* | Batch + S | Whole cells | Parr*o et a*l., 2011 |
|  | **IVE8C1** | *Acidimicrobium ferrooxidans* | Biomass from DSM Nº10331 | Whole cells | Parr*o et a*l., 2011 |
|  | **IVE8S2** | *Acidimicrobium ferrooxidans* | Biomass from DSM NL10331 | Supernatant from EDTA wash | Parr*o et a*l., 2011 |
|  | **IVE9C1** | *Leptospirillum ferrooxidans* | Batch (N2 fixing) | Whole cells (intact) | Sánchez-Garcí*a et a*l., 2018 |
|  | **IVF18C1** | *Desulfotalea psychrophila* DSM 12343 | Batch (DSMZ medium 861) | Whole cells | Riva*s et a*l., 2011 |
|  | **IVF1S1** | *Shewanella gelidimarina* | Batch (marine broth 15LC) | Culture supernatant | Riva*s et a*l., 2008 |
|  | **IVF2C1** | *Shewanella gelidimarina* | Batch (Marine broth 4ºC) | Whole cells | Riva*s et a*l., 2008 |
|  | **IVF2C2** | *Shewanella gelidimarina* | Batch (Marine broth 4LC) | Insoluble cell pellet from S100 | Riva*s et a*l., 2008 |
|  | **IVF2S100** | *Shewanella gelidimarina* | Batch (Marine broth 4ºC) | Soluble cellular fraction S100 | Riva*s et a*l., 2008 |
|  | **IVF2S2a** | *Shewanella* gelidimarina | Batch (marine broth 4LC) | Supernatant from EDTA wash | Riva*s et a*l., 2008 |
|  | **IVF31C1** | *Planococcus* sp. | Batch (TSA) | Whole cells | Parr*o et a*l., 2018 |
|  | **IVF3C2** | *Psychroserpens burtonensis* | Batch (marine broth 15LC) | Insoluble cell pellet from S100 | Riva*s et a*l., 2008 |
|  | **IVF4C1** | *Psychroserpens burtonensis* | Batch (Marine broth 4ºC) | Whole cells | Riva*s et a*l., 2008 |
|  | **IVF4S1** | *Psychroserpens burtonensis* | Batch (marine broth 4LC) | Culture supernatant | Riva*s et a*l., 2008 |
|  | **IVF4S100** | *Psychroserpens burtonensis* | Batch (Marine broth 4ºC) | Soluble cellular fraction S100 | Riva*s et a*l., 2008 |
|  | **IVF4S2a** | *Psychroserpens burtonensis* | Batch (Marine broth 4LC) | Supernatant from EDTA wash | Riva*s et a*l., 2008 |
|  | **IVF4S2b** | *Psychroserpens burtonensis* | Batch (Marine broth 4LC) | Supernatant from EDTA wash | Riva*s et a*l., 2008 |
|  | **IVF5C1** | *Psychrobacter frigidicola* | Batch (Harpo´s medium 15ºC) | Whole cells | Riva*s et a*l., 2008 |
|  | **IVF5C2** | *Psychrobacter frigidicola* | Batch (Harpo’s medium 15LC) | Insoluble cell pellet from S100 | Riva*s et a*l., 2008 |
|  | **IVF5S1** | *Psychrobacter frigidicola* | Batch (Harpo’s medium 15LC) | Culture supernatant | Riva*s et a*l., 2008 |
|  | **IVF5S100** | *Psychrobacter frigidicola* | Batch (Harpo’s medium 15LC) | Soluble cellular fraction S100 | Riva*s et a*l., 2008 |
|  | **IVF6C1** | *Cryobacterium psychrophilum* | Batch (TSA) | Whole cells | Riva*s et a*l., 2008 |
|  | **IVF6C2** | *Cryobacterium psychrophilum* | Batch (TSA) | Insoluble cell pellet from S100 | Riva*s et a*l., 2008 |
|  | **IVF6S1** | *Cryobacterium psychrophilum* | Batch (TSA) | Culture supernatant | Riva*s et a*l., 2008 |
|  | **IVF6S100** | *Cryobacterium psychrophilum* | Batch (TSA) | Soluble cellular fraction S100 | Riva*s et a*l., 2008 |
|  | **IVF6S2** | *Cryobacterium psychrophilum* | Batch (TSA) | Supernatant from EDTA wash | Riva*s et a*l., 2008 |
|  | **IVF7C1** | *Colwellia psychrerythraea* | Batch culture (Marine Broth) | Whole cells | Parr*o et a*l., 2011 |
|  | **IVF7S1** | *Colwellia psychrerythraea* | Bath culture (marine broth) | Culture supernatant | Parr*o et a*l., 2011 |
|  | **IVF7S2** | *Colwellia psychrerythraea* | Bath culture (marine broth) | Supernatant from EDTA wash | Parr*o et a*l., 2011 |
|  | **IVG1C1** | *Acidocella aminolytica* DSM 11237 | Batch (DSMZ Nº 269) | Whole cells | Parr*o et a*l., 2011 |
|  | **IVG2C_185** | *Acidiphillium* spp. | Batch culture | Whole cells | Parr*o et a*l., 2011 |
|  | **IVG2C1** | *Acidiphillium* sp. | Batch (DSMZ Nº 269) | Whole cells | Parr*o et a*l., 2011 |
|  | **IVG3C1** | *Acidobacterium capsulatum* DSM *11244* | Batch (DSMZ Nº 269) | Whole cells | Parr*o et a*l., 2011 |
|  | **IVG4C1** | *Thermus scotoductus* | Batch (TYG) | Whole cells | Parr*o et a*l., 2011 |
|  | **IVG4C2** | *Thermus scotoductus* | Batch (TYG) | Insoluble cell pellet from S100 | Parr*o et a*l., 2011 |
|  | **IVG5C1** | *Sulfobacillus acidophilus* | Biomass DSMZ No 10332 | Whole cells | Parr*o et a*l., 2011 |
|  | **IVG6C1** | *Thermus termophilus* | Batch (TYG) | Whole cells | Parr*o et a*l., 2011 |
|  | **IVH1C1** | *Bacillus subtilis* (spores) | Batch (Schaeffer medium) | Whole spores | Fernández-Calv*o et a*l., 2006 |
|  | **IVH2C1** | *Streptomyces diastaticus* (spores) | Batch (Schaeffer medium) | Whole spores | Sánchez-Garcí*a et a*l., 2018 |
|  | **IVI10C1** | *Desulfovibrio vulgaris (vulgaris)* | Biomass DSMZ No 644 | Whole cells | Riva*s et a*l., 2008 |
|  | **IVI11C1** | *Geobacter sulfurreducens* | Biomass DSMZ No 12127 | Whole cells | Riva*s et a*l., 2008 |
|  | **IVI12C1** | *Geobacter metallireducens* | Biomass DSMZ No 7210 | Whole cells | Riva*s et a*l., 2008 |
|  | **IVI13C1** | *Thermotoga maritima* | Biomass DSMZ No 3109 | Whole cells | Riva*s et a*l., 2008 |
|  | **IVI14C1** | *Verrucomicrobium spinosum* | Biomass DSMZ No 4136 | Whole cells | Riva*s et a*l., 2008 |
|  | **IVI15C1** | *Methylomicrobium capsulatum* | Biomass DSMZ No 6130 | Whole cells | Riva*s et a*l., 2008 |
|  | **IVI16C1** | *Planctomyces limnophilus* | Biomass DSMZ No 3776 | Whole cells | Riva*s et a*l., 2008 |
|  | **IVI17C1** | *Hydrogenobacter thermophilus* | Biomass DSMZ No 6534 | Whole cells | Riva*s et a*l., 2008 |
|  | **IVI19C1** | *Desulfosporosinus meridiei* | Biomass DSMZ No 13257 | Whole cells | Parr*o et a*l., 2011 |
|  | **IVI1C1** | *Pseudomonas putida* | Batch (LB) | Whole cells | Riva*s et a*l., 2008 |
|  | **IVI1C2** | *Pseudomonas putida* | Batch (LB) | Insoluble cell pellet from S100 | Riva*s et a*l., 2008 |
|  | **IVI1RB** | *Pseudomonas putida* | Batch (LB) | Ribosome fraction | Riva*s et a*l., 2008 |
|  | **IVI1S100** | *Pseudomonas putida* | Batch (LB) | Soluble cellular fraction S100 | Riva*s et a*l., 2008 |
|  | **IVI20C1** | *Salinibacter ruber* M8 | Batch (SW25 % marine salt) | Whole cells | Parr*o et a*l., 2011 |
|  | **IVI21C1** | *Salinibacter ruber* PR1 | Batch (SW25 % marine salt) | Whole cells | Parr*o et a*l., 2011 |
|  | **IVI21C2** | *Salinibacter ruber* PR1 | Batch (SW25 % marine salt) | Insoluble cell pellet from S100 | Parr*o et a*l., 2011 |
|  | **IVI21S1** | *Salinibacter ruber* PR1 | Batch (SW25% marine salt) | Culture supernatant | Parr*o et a*l., 2011 |
|  | **IVI23C1** | *Streptomyces diastaticus* (mycelium) | R2YE sporulating medium | Whole mycelium (sonicated) | Sánchez-Garcí*a et a*l., 2018 |
|  | **IVI2C1** | *Bacillus spp.* (environ. isolate Rio Tinto) | Batch (LB) | Whole cells | Riva*s et a*l., 2008 |
|  | **IVI2C2** | *Bacillus* spp. (environ. isol.) | Batch (LB) | Insoluble cell pellet from S100 | Riva*s et a*l., 2008 |
|  | **IVI2S100** | *Bacillus* spp. (environ. isol.) | Batch (LB) | Soluble cellular fraction S100 | Riva*s et a*l., 2008 |
|  | **IVI2S2** | *Bacillus* spp. (environ. isol.) | Batch (LB) | Supernatant from EDTA wash | Riva*s et a*l., 2008 |
|  | **IVI3C1** | *Shewanella oneidensis* | Batch (LB) | Whole cells | Riva*s et a*l., 2008 |
|  | **IVI3C2** | *Shewanella* oneidensis | Batch (LB) | Insoluble cell pellet from S100 | Riva*s et a*l., 2008 |
|  | **IVI3S100** | *Shewanella* oneidensis | Batch (LB) | Soluble cellular fraction S100 | Riva*s et a*l., 2008 |
|  | **IVI3S2** | *Shewanella* oneidensis | Batch (LB) | Supernatant from EDTA wash | Riva*s et a*l., 2008 |
|  | **IVI4C1** | *Burkholderia fungorum* | Batch (LB) | Whole cells | Riva*s et a*l., 2008 |
|  | **IVI4C2** | *Burkholderia fungorum* | Batch (LB) | Insoluble cell pellet from S100 | Riva*s et a*l., 2008 |
|  | **IVI4S100** | *Burkholderia fungorum* | Batch (LB) | Soluble cellular fraction S100 | Riva*s et a*l., 2008 |
|  | **IVI4S2** | *Burkholderia fungorum* | Batch (LB) | Supernatant from EDTA wash | Riva*s et a*l., 2008 |
|  | **IVI5C1** | *Shewanella oneidensis* | Anaerobic (fumarate) | Whole cells | Riva*s et a*l., 2008 |
|  | **IVI5C2** | *Shewanella* oneidensis | Anaerobic (fumarate) | Insoluble cell pellet from S100 | Riva*s et a*l., 2008 |
|  | **IVI5S1** | *Shewanella* oneidensis | Anaerobic (fumarate) | Culture supernatant | Riva*s et a*l., 2008 |
|  | **IVI5S100** | *Shewanella* oneidensis | Anaerobic (fumarate) | Soluble cellular fraction S100 | Riva*s et a*l., 2008 |
|  | **IVI6C3** | *Azotobacter vinelandii* | Batch culture (LB) | EDTA washed cells (sonicated) | Riva*s et a*l., 2008 |
|  | **IVI7C1** | *Bacillus subtilis 168* | Batch culture (LB) Vegetative cells | Whole cells | Riva*s et a*l., 2008 |
|  | **IVI8C1** | *Bacillus subtilis* 3610 | Biofilm | Whole cells | Riva*s et a*l., 2008 |
|  | **IVI8S1** | *Bacillus subtilis* 3610 | Biofilm | Culture supernatant | Riva*s et a*l., 2008 |
|  | **IVI9C1** | *Deinococcus radiodurans* | Biomass DSMZ No 20539 | Whole cells | Riva*s et a*l., 2008 |
|  | **IVJ1C1** | *Haloferax mediterranei* | Batch (SW25 % marine salt) | Whole cells | Riva*s et a*l., 2008 |
|  | **IVJ2C1** | *Methanococcoides burtonii* | Biomass DSMZ No 6242 | Whole cells | Riva*s et a*l., 2008 |
|  | **IVJ3C1** | *Thermoplasma acidophilum* | Biomass DSMZ No 1728 | Whole cells | Riva*s et a*l., 2008 |
|  | **IVJ4C1** | *Methanobacterium formicicum* | Biomass DSMZ No 1535 | Whole cells | Riva*s et a*l., 2008 |
|  | **IVJ5C1** | *Methanosarcina mazeii* | Biomass DSMZ No 3647 | Whole cells | Riva*s et a*l., 2008 |
|  | **IVJ6C1** | *Pyrococcus furiosus* | Biomass DSM No 3638 | Whole cells | Parr*o et a*l., 2011 |
|  | **IVJ8C1** | *Halorubrum* sp*.* | Batch (SW25 % marine salt) | Whole cells | Parr*o et a*l., 2011 |
|  | **IVJ9C1** | *Halobacterium* sp*.* | Batch (SW25 % marine salt) | Whole cells | Parr*o et a*l., 2011 |
|  | **IVK1C1** | *Anabaena* sp. PCC7120 | BG11 and nitrate | Whole cells | Blanc*o et a*l., 2015 |
|  | **IVK1S2** | *Anabaena* sp. PCC7120 | BG11 and nitrate | Supernatant from EDTA wash | Blanc*o et a*l., 2015 |
|  | **IVK2C1** | *Anabaena* sp. PCC7120 | BG11o | Whole cells | Blanc*o et a*l., 2015 |
|  | **IVK2S2** | *Anabaena* sp. PCC7120 | BG11o | Supernatant from EDTA wash | Blanc*o et a*l., 2015 |
|  | **p-139** | Pre-immune serum | IgG fraction (protein A purified) | Pre-immune serum | Riva*s et a*l., 2008 |
|  | **p-1496** | Pre-Immune serum | IgG fraction (protein A purified) | Pre-Immune serum | Riva*s et a*l., 2008 |
|  | **p-ABCtrans** | Pre-Immune serum | IgG fraction (protein A purified) | Pre-Immune serum | Parr*o et a*l., 2011 |
|  | **p-CrReTs_977** | Pre-immune serum | IgG fraction (protein A purified) | Pre-immune serum | Parr*o et a*l., 2011 |
|  | **p-FeReTs_983** | Pre-immune serum | IgG fraction (protein A purified) | Pre-immune serum | Parr*o et a*l., 2011 |
|  | **p-IVE1C1** | Pre-immune serum | IgG fraction (protein A purified) | Pre-immune serum | Riva*s et a*l., 2008 |
|  | **p-IVE3C1** | Pre-immune serum | IgG fraction (protein A purified) | Pre-immune serum | Riva*s et a*l., 2008 |
|  | **p-IVE4C1** | Pre-immune serum | IgG fraction (protein A purified) | Pre-immune serum | Riva*s et a*l., 2008 |
|  | **p-IVE5C1** | Pre-immune serum | IgG fraction (protein A purified) | Pre-immune serum | Riva*s et a*l., 2008 |
|  | **p-IVE6C1** | Pre-immune serum | IgG fraction (protein A purified) | Pre-immune serum | Riva*s et a*l., 2008 |
|  | **p-IVF2C1** | Pre-immune serum | IgG fraction (protein A purified) | Pre-immune serum | Riva*s et a*l., 2008 |
|  | **p-IVG2C1** | Pre-Immune serum | IgG fraction (protein A purified) | Pre-Immune serum | Parr*o et a*l., 2011 |
|  | **p-IVG4C1** | Pre-immune serum | IgG fraction (protein A purified) | Pre-immune serum | Parr*o et a*l., 2011 |
|  | **p-IVI10C1** | Pre-immune serum | IgG fraction (protein A purified) | Pre-immune serum | Parr*o et a*l., 2011 |
|  | **p-IVI12C1** | Pre-immune serum | IgG fraction (protein A purified) | Pre-immune serum | Sánchez-Garcí*a et a*l., 2018 |
|  | **p-IVI20C1** | Pre-Immune serum | IgG fraction (protein A purified) | Pre-Immune serum | Parr*o et a*l., 2011 |
|  | **p-IVI2C1** | Pre-immune serum | IgG fraction (protein A purified) | Pre-immune serum | Riva*s et a*l., 2008 |
|  | **p-IVI8C1** | Pre-immune serum | IgG fraction (protein A purified) | Pre-immune serum | Riva*s et a*l., 2008 |
